# Supplementary material for: Chromothripsis during telomere crisis is independent of NHEJ, and consistent with a replicative origin
Source: Genome Res. 2019 May;29(5):737–49. doi: 10.1101/gr.240705.118 (PMC6499312; doi:10.1101/gr.240705.118)
Supplement: Supplemental Material [file supp_gr.240705.118_Supplemental_file_1.zip › contigs/annotated_contigs/DB50/contig.2.DB50_length_471_mean_cov_6.23566878981.docx]

**DB50_length_471_mean_cov_6.23566878981**

```
TCATTGACACACACTCATGCCTTCGTCACATAATCAAAGTATGAGAGCATTTTGGTCATTAGAGTAGCGGCCAGTAGAGTAAGATGAAT
 >chr10:7326443-7326644 - E=2e-106
GTCTGGGAAACCGTCATGGTGTAAATTGAATGCTCTGTATTAGAATTATCTGTCTGTTGGAAAAGTAGACCGTTTGCTACCACGTTTTC

TAGGTTCAGGCAAATTTGGGTAG|G|TGTGACGAGGGAACGTTCGGCTACTCCTGGAAGATTGATGGGCTCCATCAGCAACCCACAGCC
                         >chr10:1728518-1728765 - E=4e-134
TTGTTTCTTATCACGCAACCACCATTAGACCTTCCCCTACTTTCACACAAATTAACTGCTCATCAGTTTCCTGATTGCAAGAGCTTGCG

GCAGCTGTAGTTTTCAAGGTATGCTTCCTTCAAACCTATTTGGTTCTCACTCTCCCAGCACATGATCCTGTCCTTGTTCGACTCAATTG

ATTCAT|GCGAACCGGTGGAGGACCGAGC
```
